# Pediatric Anterior Cruciate Ligament Reconstruction: A Reason Not to Wait Until Skeletal Maturity

**DOI:** 10.7759/cureus.19597

**Published:** 2021-11-15

**Authors:** Benjamin T Harris, Elizabeth A Eichman, Manraj J Johal, Matthew T Burrus

**Affiliations:** 1 College of Osteopathic Medicine, University of New England, Biddeford, USA; 2 School of Medicine, Texas Tech University Health Sciences Center, Lubbock, USA; 3 Department of Orthopedic Surgery, Orthopedic Associates of Central Texas, Austin, USA

**Keywords:** meniscus tear, meniscus, pediatrics, acl injury, orthopedic sports medicine

## Abstract

Anterior cruciate ligament (ACL) tears within the skeletally immature population give rise to controversy regarding the timing of treatment decisions due to the concern of iatrogenic damage to the open physis. Physis disruption from the required intraoperative graft tunnel drilling can lead to growth disturbance, thus ligament reconstruction is not without risk. Nonoperative management carries the risk of future damage to the menisci and cartilage as an ACL-deficient knee can be unstable. This particular case of a skeletally immature 10-year old male demonstrates an initial course of nonoperative treatment which ultimately resulted in previously undiagnosed meniscal damage. Failure of the nonoperative treatment was followed by a successful ACL reconstruction and meniscal repair surgery utilizing a partial physeal sparing technique. The patient successfully returned to his preoperative activity level without any graft disruption, postoperative indications of meniscus pathology, or abnormal growth deformities. This case report adds to the current literature reporting successful and safe ACL reconstructions in a skeletally immature patient.

## Introduction

A tear of the anterior cruciate ligament (ACL) is a devastating injury to the knee joint that could affect the patient’s long-term quality of life. A concerning trend is an increase in the incidence of pediatric ACL tears. From 1994 to 2013 there was a reported 2.3% annual increase in pediatric ACL tears [1]. Reconstruction rates have increased as well, in 1990 the rate of ACL reconstruction was 17.6 per 100,000 patients aged three to 20 years which increased to a rate of 50.9 reconstructions in 2009 [2]. The increase in pediatric ACL tears is thought to be due to the increased number of athletes, year-round sports participation, and single-sport specialization [3]. An ACL tear at a young age is especially concerning due to the longer duration of life to live compared to the average adult, increased likeliness to be involved in multiple sports, and the potential onset of post-traumatic osteoarthritis earlier in life [4,5].

Historically, initial treatment of an ACL tear in a skeletally immature patient includes nonoperative modalities [6]. Nonoperative treatment usually consists of bracing the knee, physical therapy, and activity modification. The rationale behind the nonoperative treatment in a skeletally immature patient is to allow the patient to achieve skeletal maturity before performing ACL reconstruction in order to minimize the risk of iatrogenic growth disturbance. The standard ACL reconstruction technique creates graft tunnels by removing a cylindrical tube of bone. Thus, in a skeletally immature patient drilling the graft tunnels introduces a risk for disruption of the growth plates. However, there are alternate surgical techniques that create graft tunnels drilled in a manner that minimizes the risk of disturbance to the growth plate.

This case demonstrates an ACL tear in a skeletally immature patient who was initially treated nonoperatively. Later, the patient developed a previously unidentified meniscal tear. Ultimately, the patient underwent reconstruction using an all-epiphyseal tunnel on the femur and a transphyseal tunnel on the tibia without postoperative complication.

## Case presentation

A healthy 10-year-old male presented to primary care with left knee pain after hearing a pop in his knee while falling off a dirt bike. He had no history of prior knee injury or pertinent past medical, surgical, or family history. His exam and history resulted in radiography, magnetic resonance imaging (MRI), and referral to an orthopedic specialist.

The radiographs demonstrated open physis, effusion, and no fractures. MRI revealed an ACL tear, intact menisci, and lateral compartment pivot-shift edema. Neither the radiologist nor the surgeon appreciated any medial or lateral meniscus pathology on the MRI. The physical examination of the knee included moderate effusion, no varus or valgus instability, 2B Lachman, and a positive pivot-shift test. Lachman exams are graded one (3-5 millimeters [mm] of translation), two (5-10 mm of translation), or three (greater than 10 mm of translation) based on the amount of anterior translation of the tibia. The exam is further classified by whether the endpoint feels firm (A) or soft (B). The examination was not consistent with meniscal pathology. Conservative treatment consisting of an ACL hinged knee brace, physical therapy, activity modification, and nonsteroidal anti-inflammatory drugs was recommended to determine if the knee could be stabilized until the patient attained skeletal maturity.

**Figure 1:**
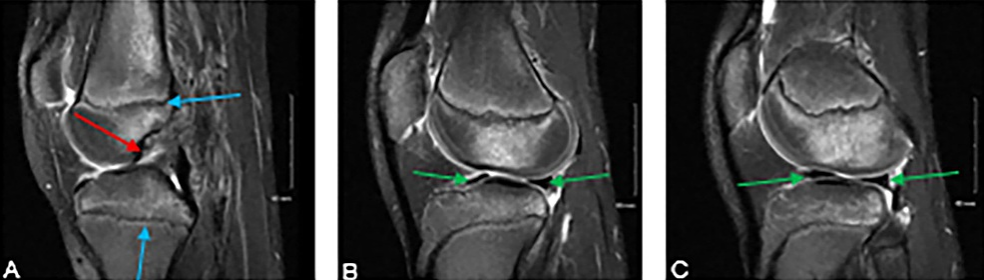
T-2 weighted MRI sequences of the left knee demonstrating an isolated ACL tear (A) (red arrow). Medial (B) and lateral (C) menisci appear to be intact (green arrows). The open physis are identified with blue arrows.

Two years later, the patient presented to the attending surgeon following repeated instability events. Repeat radiographs and MRI were obtained. Radiographs demonstrated an open physis. MRI indicated an ACL tear and intact menisci; the pertinent images can be seen in Figures 1A-1C. The physical examination consisted of a 2B Lachman, mild to moderate lateral joint line tenderness, and negative medial and lateral McMurray’s. Based on the recurrent instability events and risk for continued intra-articular damage operative intervention was recommended. Continued nonoperative management was discussed but not recommended due to the demonstrated instability of the knee.

**Figure 2:**
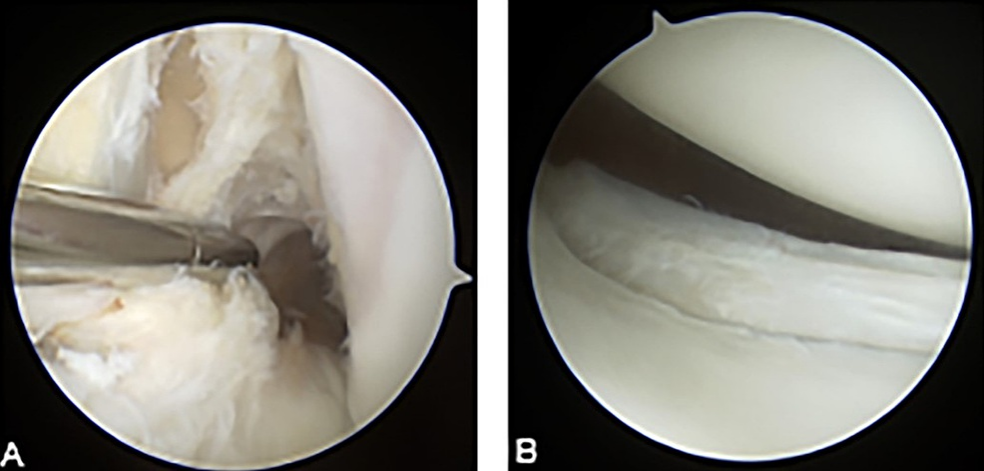
Intraoperative arthroscopic images of a complete ACL tear (A) and previously unidentified unstable tear of the posterior horn of the lateral meniscus (B).

Intraoperatively, the preoperative diagnosis of an ACL tear was confirmed as seen in Figure 2A. A previously unidentified vertical, full-thickness lateral meniscus tear that extended from the posterior root to the popliteal hiatus in the red-white junction was discovered (Figure 2B). Due to the age of the patient and the size and location of the tear, the meniscus was repaired. The repair consisted of utilizing a rasp to roughen up the synovial surface as well as the tear itself followed by all-inside meniscal repair devices (Fast-Fix Smith & Nephew, London, UK) deployed in a vertical-mattress suture configuration.

**Figure 3:**
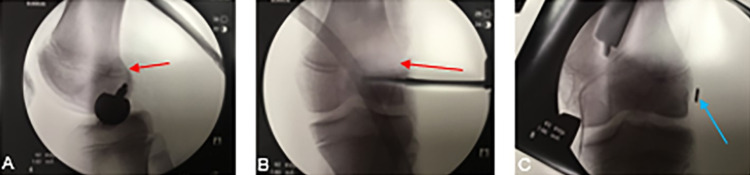
Lateral (A) and anterior-posterior (B, C) intraoperative fluoroscopic imaging used to confirm the accurate location of the femoral tunnel. The tunnel was placed as low and as posteriorly as possible, due to the open physis (red arrows). Suspensory fixation was utilized on the femur and is identified by the blue arrow (C).

**Figure 4:**
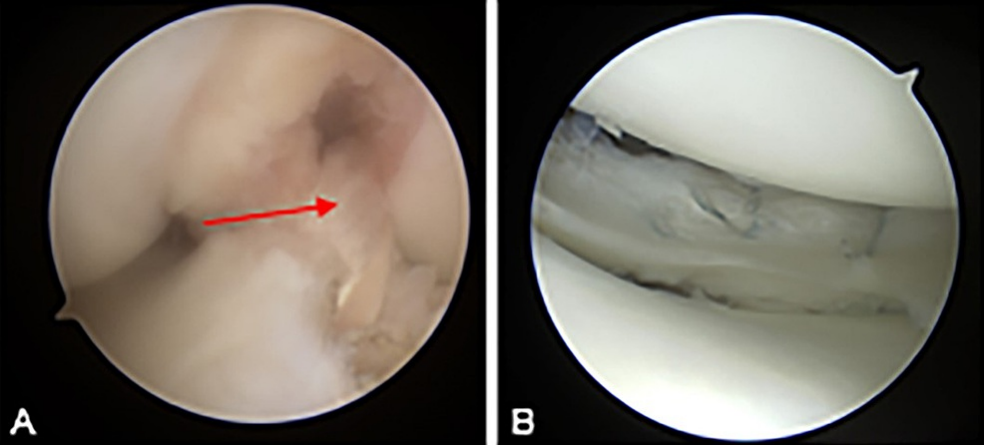
Intraoperative arthroscopic imaging of the reconstructed ACL (A) with hamstring autograft (red arrow). Intraoperative image (B) of the arthroscopic lateral meniscus repair.

The ACL reconstruction was performed using a partial physeal-sparing technique. A soft tissue hamstring autograft was created using the gracilis and semitendinosus tendons. An all-epiphyseal technique on the femur and a more posteriorly placed transepiphyseal tibial tunnel were used. Regarding the femoral tunnel, a pediatric femoral ACL guide was used to place a transverse femoral tunnel staying as low and as posteriorly as possible. Anterior-posterior and lateral fluoroscopic images were taken to confirm a tunnel location distal to the femoral epiphysis and can be seen in Figures 3A-3C. A retrograde, inside-out reamer was then used to create a tunnel 20 mm in depth and 7.5 mm in diameter. Regarding the tibial tunnel, a cannulated acorn reamer created a 9 mm tunnel with the tunnel aperture being slightly posterior to the posterior border of the anterior horn of the lateral meniscus. Suspensory fixation was used on the femoral aspect and a bio-composite interference screw (Arthrex, Naples, FL) was used on the tibial side. After final tightening, the Lachman exam had normalized. The reconstructed ACL and repaired lateral meniscus can be seen in Figures 4A, 4B.

Follow up

Postoperatively, the patient was non-weight bearing with the left knee range of motion restricted to 0-70 degrees for the first two weeks and then advanced to 0-90 degrees for four weeks to promote healing of the meniscal repair. After which he followed the standard ACL reconstruction recovery protocol. Throughout the recovery process, there were no indications of a nonhealing or recurrent meniscal tear evidenced by a lack of joint line tenderness and a negative McMurray’s test. At nine months postoperative he was cleared for a return to sport. At 36 months postoperative, no growth plate disturbances were appreciated on radiographs and there was no clinical evidence suggesting recurvatum or valgus deformity.

## Discussion

In the past, and in this case, complete ACL tears in a skeletally immature patient were initially treated with nonoperative treatment, delaying reconstruction until skeletal maturity was attained. This course of treatment usually included a combination of physical therapy, bracing, and activity modification. However, delaying reconstruction subjects the patient to possible incidences of knee instability as the ACL stabilizes the knee in the sagittal and rotational planes [7]. An ACL deficient knee has shown to be at an increased risk for intra-articular damage such as meniscal tears and chondral lesions secondary to knee instability [8,9]. Due to the significant association between meniscal damage and the development of radiographic osteoarthritis, treatment methods that may prevent meniscal damage can be preferable [10].

Nonoperative treatment may result in meniscal damage in this patient population because ACL knee braces, even though commonly prescribed, do not effectively prevent instability. Smith et al. found that the biomechanical and clinical evidence on functional bracing does not support the current wide use of bracing an ACL deficient knee [11]. Even with bracing and a physical therapy program, active athletes were not often successfully treated nonoperatively, as significant activity modification is required [12].

A rationale for pursuing initial nonoperative treatment of an ACL tear in a skeletally immature patient includes the fear of growth abnormalities such as leg-length discrepancies or axis deviations that can occur after ACL reconstruction. In a survey of the Herodicus Society, investigators found that risk factors associated with growth disturbances after ACL reconstruction included large transphyseal tunnels, lateral extra-articular tenodesis, and suturing near the tibial tubercle [13]. Additionally, 8-mm diameter tibial tunnels have been found to occupy 3.5% of the physis [14], and tunnels that occupy 7%-9% of the physis are at risk for causing growth disturbances [15]. The transphyseal tibial tunnel was 9 mm in this case. Due to the risk of physeal closure on the tibia, the transphyseal tunnel was made more posteriorly (and thus centrally located) to avoid causing a peripheral physeal arrest and angular deformity. More centrally located tibial tunnels have previously been studied and produced high success rates with low morbidity [16].

Overall, ACL reconstruction in a skeletally immature patient is a safe procedure that has shown significant improvement over time. The predicted risk of growth abnormalities has decreased from 4.2% in 1982 to 1.1% in 2009. Further, the likelihood of achieving an International Knee Documentation Committee (IKDC) grade of A or B increased from 67.4% in 1982 to 88.5% in 2009 [17]. The rates of growth abnormalities occurring with a physeal-sparing technique have been reported at 5.8% (n=139) with reruptures occurring 1.4% of the time [17]. Not only is ACL reconstruction in the skeletally immature safe and effective, but also patients who undergo early instead of delayed reconstruction have demonstrated clinically superior outcomes assessed by the IKDC score (94.6 vs 82.4) [18]. This may be because the time to surgery is greater than three months is significantly associated with medial meniscal tears in a pediatric population awaiting ACL reconstruction [8]. The mean clinical important difference between IKDC scores is nine points [19]. Thus, early reconstruction provided a clinically significant improvement in outcome when compared to delayed reconstruction.

## Conclusions

It is recommended for orthopedic surgeons to consider the risk and benefits of nonoperative versus operative treatment in the setting of an ACL tear in skeletally immature patients. Clinical examination, imaging, and the patient’s activity level are essential factors in determining the best course of action. Reconstruction in this case offered the patient an excellent outcome without postoperative complications. However, the initial course of nonoperative treatment likely resulted in meniscal damage that could have potentially been prevented if surgical treatment occurred earlier.
